# The mechanism of berberine alleviating metabolic disorder based on gut microbiome

**DOI:** 10.3389/fcimb.2022.854885

**Published:** 2022-08-25

**Authors:** Han Wang, Haiyu Zhang, Zezheng Gao, Qiqi Zhang, Chengjuan Gu

**Affiliations:** ^1^ School of Chinese Materia Medica, Beijing University of Chinese Medicine, Beijing, China; ^2^ Guang’anmen Hospital, China Academy of Chinese Medical Sciences, Beijing, China; ^3^ Shenzhen Hospital (Futian), Guangzhou University of Chinese Medicine, Shenzhen, China

**Keywords:** gut microbiota, metabolic disorder, diabetes, obesity, berberine

## Abstract

With socioeconomic advances and improved living standards, metabolic syndrome has increasingly come into the attention. In recent decades, a growing number of studies have shown that the gut microbiome and its metabolites are closely related to the occurrence and development of many metabolic diseases, and play an important role that cannot be ignored, for instance, obesity, type 2 diabetes (T2DM), non-alcoholic fatty liver disease (NAFLD), cardiovascular disease and others. The correlation between gut microbiota and metabolic disorder has been widely recognized. Metabolic disorder could cause imbalance in gut microbiota, and disturbance of gut microbiota could aggravate metabolic disorder as well. Berberine (BBR), as a natural ingredient, plays an important role in the treatment of metabolic disorder. Studies have shown that BBR can alleviate the pathological conditions of metabolic disorders, and the mechanism is related to the regulation of gut microbiota: gut microbiota could regulate the absorption and utilization of berberine in the body; meanwhile, the structure and function of gut microbiota also changed after intervention by berberine. Therefore, we summarize relevant mechanism research, including the expressions of nitroreductases-producing bacteria to promote the absorption and utilization of berberine, strengthening intestinal barrier function, ameliorating inflammation regulating bile acid signal pathway and axis of bacteria-gut-brain. The aim of our study is to clarify the therapeutic characteristics of berberine further and provide the theoretical basis for the regulation of metabolic disorder from the perspective of gut microbiota.

## 1. Introduction

Metabolic disorders are a complex group of multifactorial disorders formed by one or more causes of glucose metabolism, lipid metabolism, purine metabolism and so on, including central obesity, insulin resistance, abnormal glucose metabolism, lipid metabolism disorders, non-alcoholic fatty liver disease(NFLD), and metabolic hypertension, among others (Kassi et al., 2011). With the improvement of living standards, the incidence of metabolic disorder is also increasing. The existence of multiple metabolic disorders not only increases the incidence rate of diabetes directly, but also greatly increases the risk of atherosclerotic cardiovascular disease, brings enormous burden to the global medical and health field (2001). Insulin resistance is the core factor of metabolic syndrome, it is also a common pathological manifestation of metabolic disorders such as diabetes and obesity (Balkau and Charles, 1999; Huang, 2009). The current situation shows that a large proportion of metabolic syndrome patients suffer from impaired glucose tolerance or diabetes (Shinkov et al., 2018).

The relationship between the occurrence of metabolic disorders with the gut microbiota is one of the main research directions to explore the mechanism of metabolic disorders at present (Meijnikman et al., 2018). The human gut flora is a very complex system of a great variety, according to current reports, more than 1000 kinds of microorganisms in the human gastrointestinal tract are known, which belong to five phyla: *Bacteroidetes, Firmicutes, Actinobacteria, Proteobacteria*, and *Verrucomicrobia (* Wu et al., 2021). Among which, anaerobic *Bacteroides* and *Firmicutes* are the two dominant species, accounting for more than 90% of all bacterial species (Qin et al., 2010). Numerous studies have shown that alterations in the composition of the gut microbiota have been associated with metabolic disorders (Festi et al., 2014; Ussar et al., 2015), including obesity (Gomes et al., 2018), DM (Baothman et al., 2016; Yang et al., 2021), high fat diet (Yin et al., 2018; Leeming et al., 2019; Schoeler and Caesar, 2019), antibiotics (Ianiro et al., 2016; Ramirez et al., 2020), and other factors can lead to gut microbiota dysbiosis (Jin et al., 2017; Greenhill, 2018; Guerre, 2020; Philippe, 2020), and at the same time, the gut microbiota can be mediated by drugs that regulate the body’s metabolism and play a role in improving metabolic disorders (Feng et al., 2020).

Consistently, as a natural medicine, berberine plays an important role in the treatment of metabolic disorders. Its mechanism of utility has also been extensively and intensively studied (Caliceti et al., 2016), a large number of experimental studies have shown that berberine alleviates body metabolism and insulin resistance through a variety of mechanisms, such as amelioration of oxidative stress, inhibition of macrophage inflammatory response, adenosine monophosphate-activated protein kinas (AMPK) activation and Phosphorylation of acetyl-CoA carboxylase (ACC), regulating mitochondria-related pathways (Zhang et al., 2018), inducing peroxisome proliferator-activated receptors (PPARs) increase^6^Modulating transcriptional programs of transcription factors, among others (Wang et al., 2020). In recent years, the mechanism of berberine in treating metabolic disorders by modulating gut microbiota has been increasingly discovered and recognized. In this paper, the author focuses on the mechanism of BBR ameliorate metabolic disorder, so as to further clarify the therapeutic characteristics of BBR, providing a theoretical basis for regulating and alleviating metabolic disorder from the perspective of gut microbiome.

## 2.Berberine for metabolic disorders

**Figure 1:**
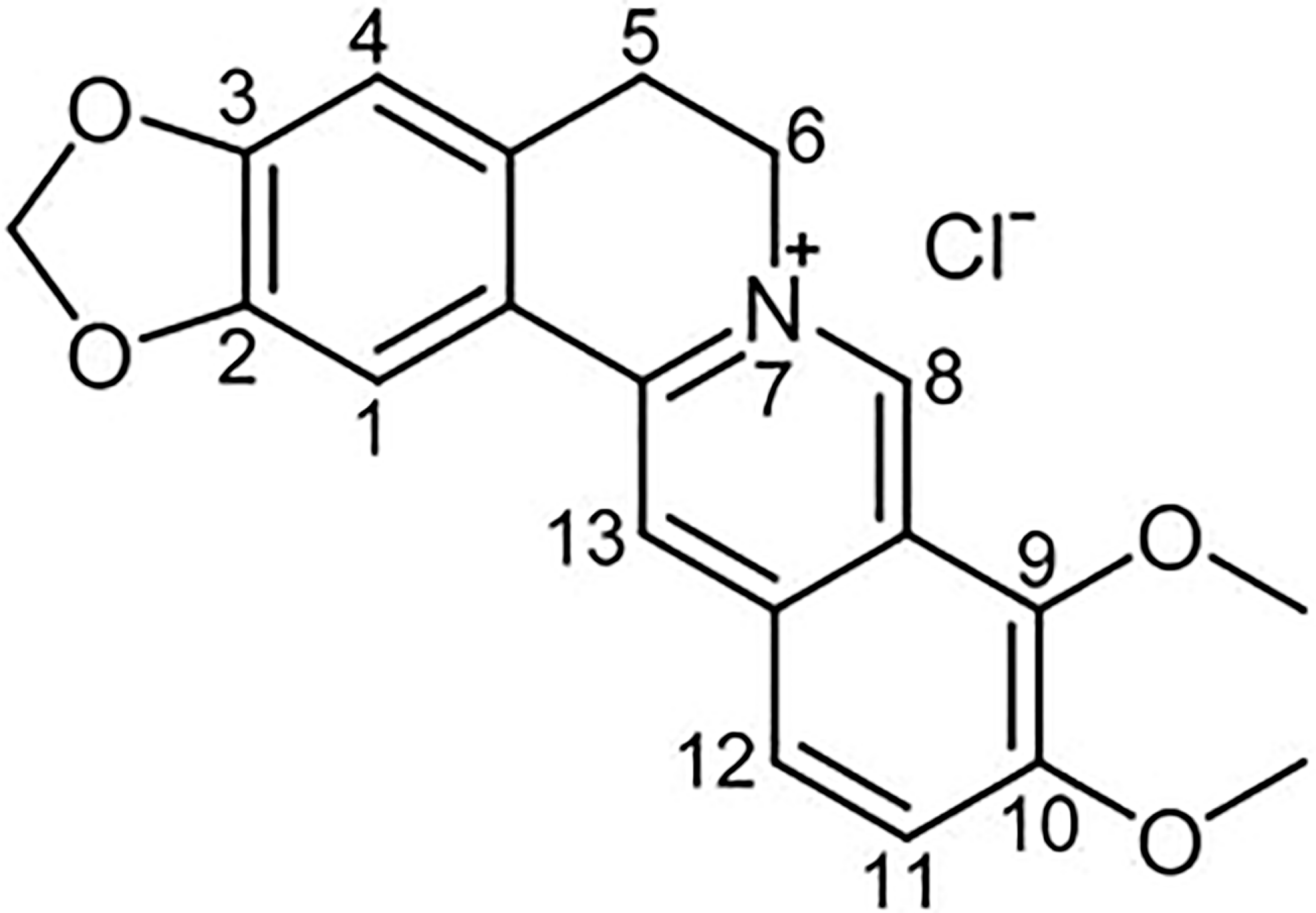
Chemical structural formula of Berberine (BBR).

Berberine (BBR) is a quaternary ammonium salt from the group of bioactive isoquinoline alkaloids (Imenshahidi and Hosseinzadeh, 2016), which is the major active component of traditional Chinese herb Coptis chinensis (Tabeshpour et al., 2017). In addition, BBR also exist in various medicinal plants such as *Berberis aristata*, *B. petiolaris*, *B. aquifolium*, *B. vulgaris*, *B. thunbergia* and many others (Cicero and Baggioni, 2016; Liu et al., 2019). Its chemical structure is represented in **Figure 1**. In recent years, a large number of research confirmed that BBR has extensive pharmacological properties, such as anti-inflammatory (Kuo et al., 2004), pain‐relieving (Hashemzaei and Rezaee, 2021), anti-infective (Aswathanarayan and Vittal, 2018), antitumor (Liu et al., 2019),neuroprotective (Luo et al., 2012) and modulate energy metabolism, which can be used to treat cancer (Zhang et al., 2020), digestive (Zou et al., 2017), metabolic (Xu et al., 2021), cardiovascular (Martini et al., 2020), and neurological diseases (Kulkarni and Dhir, 2010). Formulas containing traditional herbal Coptis chinensis, has been used for thousands of years in traditional Chinese Medicine. Most of these formulas possess the efficacy of clearing heat, eliminating dampness, dissipating fire and detoxifying (Wang et al., 2017), similar to what we now considered as anti-infective and anti-inflammatory functions.

Accumulating clinical studies have shown that BBR has a good role in the treatment of metabolic diseases such as diabetes, obesity, non-alcoholic fatty liver disease and hyperlipidemia. BBR could ameliorate insulin resistance in patients with diabetes, lower blood sugar and hemoglobin A1c (HbA1c) in patients with T2DM (Yin et al., 2008; Zhang et al., 2010). Obesity is closely related to an increased risk of T2DM, BBR could successfully decrease body weight, body mass index, waist circumference in obese patients with T2DM (Xu et al., 2021). In addition, BBR can improve the hepatic fat content, apolipoprotein B (Apo B), alanine aminotransferase (ALT), and aspartate aminotransferase (AST) in patients with NAFLD (Yan et al., 2015). Furthermore, BBR treatment can substantial reduction in TC, TG, and LDL-C levels in patients with Hyperlipidemia (Kong et al., 2004).

In addition to clinical studies, berberine has been extensively explored in the treatment of metabolic syndrome in terms of mechanisms. Berberine has many extensive and intensive studies in the field of metabolic diseases. It can regulate and ameliorate glycolipid metabolism (Ma et al., 2018; Yu et al., 2019; Bertuccioli et al., 2020), lose weight (Neyrinck et al., 2021) and alleviation of insulin resistance (Memon et al., 2018), especially in the treatment of combined multiple metabolic disorders, the efficacy of BBR is outstanding (Yu-Guang et al., 2017). Meanwhile, numerous experimental studies have shown that BBR ameliorate body metabolic dysfunction through multiple mechanisms (Yin et al., 2012; Ilyas et al., 2020). In terms of glucose metabolism, BBR strengthened autophagy and protected from high glucose-related injury in podocytes by promoting the AMPK activation, at the same time, the bidirectional regulation of AMPK activity can reduce the risk of hypoglycemia caused by berberine (Xiao et al., 2018). BBR also through TLR4/MyD88/NF-κB signaling pathway improves gut-derived hormones (Gong et al., 2017a), alleviate inflammation by reducing the exogenous antigen load in the host (Zhang et al., 2012). In the regulation of blood lipids, BBR can decrease triglyceride expression in HepG2 cell lines (Cao et al., 2018), modulation of bile acids (He et al., 2016a) and upregulation of Trib1 mRNA levels to reduce lipid levels (Singh and Liu, 2019). With further in-depth studies scholars have found that modulation of gut microbiome may be another novel mechanism of action for berberine to ameliorate metabolic disorders (Hvistendahl, 2012; Feng et al., 2015).

## 3. Impact of gut microbiota on metabolic disorders

Earlier studies have shown that alterations in the gut microbiota are environmental factors that influence host susceptibility to obesity and increase adiposity (Backhed et al., 2004). When feces from obese and lean pairs of twins were transplanted separately into germ free mice, mice transplanted with feces from obese recipients gained weight and exhibited an obesity associated metabolic phenotype, whereas mice transplanted with feces from lean recipients showed no significant metabolic phenotype (Ridaura et al., 2013). Therefore, gut microbiota is strongly associated with the occurrence of metabolic disorders.

On the one hand, the findings suggest that metabolic disorders significantly affect the structure and function of the gut microbiota. On the other hand, gut microbiota can produce metabolites by fermenting dietary fiber, for instance, short-chain fatty acids (SCFAs) and bile acids (BAs), have important metabolic functions (Canfora et al., 2019), can significantly affect body metabolism function. The *Firmicutes/Bacteroidetes* (F/B) ratio is widely accepted to have an important influence in maintaining normal intestinal homeostasis, the increase of F/B will lead to obesity (Stojanov et al., 2020). Numerous studies have confirmed that, humans or animals with obesity/insulin resistance or diabetes have an elevated abundance of opportunistic pathogens (such as sulfate reducing bacteria) and a decreased abundance of beneficial bacteria (as *F.prausnitzii* (Xu et al., 2020a), *A.muciniphila*, *R.intestinalis, Bifidobacterium and Akkermansia muciniphila.*) in the gut (Everard et al., 2011; Qin et al., 2012; Tilg and Moschen, 2014; Feng et al., 2015; Cani, 2019). Further studies revealed that certain specific bacteria have important effects on body metabolism, such as the endotoxin producing bacterium *E.clocae* isolated from obese patients, which can cause obesity and insulin resistance in axenic mice (Fei and Zhao, 2013). It follows that the metabolic function of the organism interacts with the gut microbiome, and modulation of the gut microbiota can improves metabolic function.

In recent years, a large number of studies have focused on the relationship between berberine and gut microbiota. Research shows can ameliorate metabolic abnormalities by modulating the flora to enhance bioavailability (Liu et al., 2016), regulating the bacteria-brain-gut axis (Sun et al., 2016; Sun et al., 2017), strengthening intestinal barrier function (Zhang et al., 2012; Gong et al., 2017a), alleviating metabolic endotoxemia (Gao et al., 2017), increasing of short-chain fatty acid (SCFA)-producing bacteria (Mcnabney and Henagan, 2017) and other ways. Now, these mechanisms will be systematically summarized and elaborated in this review.

## 4. Mechanism of berberine ameliorates metabolic disorders based on gut microbiome

**Figure 2:**
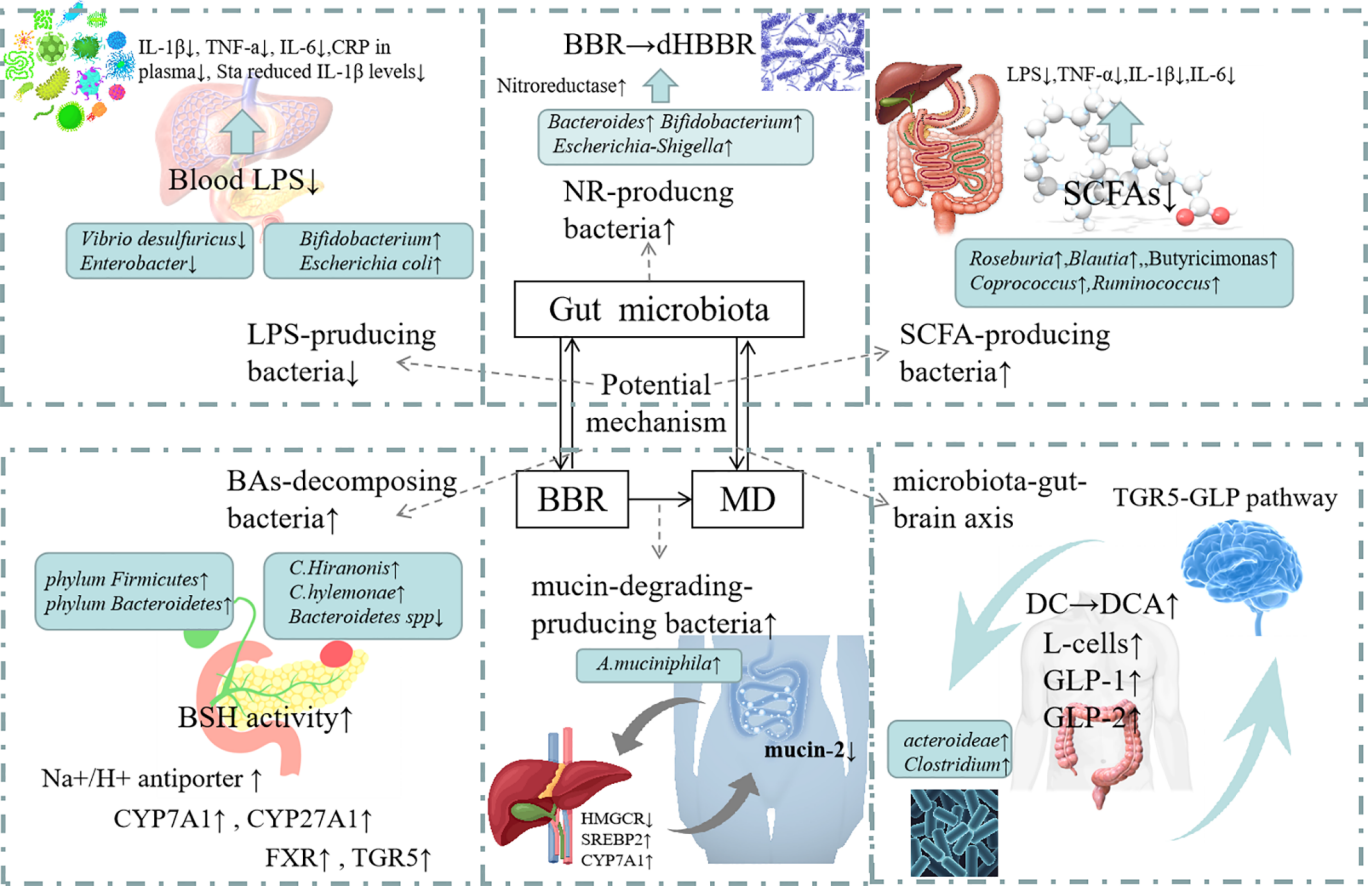
Mechanism of action of Berberine in modulating Gut Microbioata. Increasing of the NR-producing bacteria: BBR can increase th abundance of *Bacteroides*, *Escherichia-Shigella* and *Bifidobacterium* which can produc NRs. Nrs converts BBR into its absorbable form of DhBBR, which has highly polar and easily absorbed into the blood. Increasing of mucin-degrading-producing bacteria: BBR can increase the abundance of *A.muciniphila*. The increased abundance of *A.muciniphila* may lead to the reduction in mucin-2 expression in ileum. BBR seemed to protect the intestinal barrier integrity through modulating HMGCT, SREBP2 and CYP7A1 expressions. Decreasing of LPS-producing bacteria: BBR reduced the level of *Vibrio desulfuricus* and *Enterobacter* to inhibit the production of LPS. So that inflammatory factors (IL-1β, TNF-a IL-6, CRP in plasma Sta reduced IL-1β levels) were decreased. Increasing of SCFA-producing bacteria: BBR increased the number of SCFA producing bacteria (such as *Butyricimonas*, *Coprococcus*, *Ruminococcus* and *Roseburia*), raised the expression of pro-inflammatory cytokines, including LPS, TNF-α IL-1β and IL-6. Increasing of BAs-decomposing bacteria: BBR increased the number of BAs-decomposing bacteria (such as *phylum Firmicutes*, *phylum Bacteroidetes*, *C. scindens* and *C. hylemonae*) and reduced the level of *C. hiranosis*, decreased the activity of BSH. The possible mechanism is to Up-regulate Na+/H+ antiporter, up-regulate colonic TGR5 expression and GLP secretion and to increase CYP7A1 and CYP27A1 expression. Regulating microbiota-gut-brain axis: BBR increases the propotion of *Bacteroidetes* and *Firmicutes*, increases the expression of serum GLP-1, GLP-2, increase of the number of L.cells.

Berberine can induce cell death of harmful intestinal bacteria and increase the number and species of beneficial bacteria (Habtemariam, 2020). From administration to absorption, BBR can be metabolized by gut microbiota to improve its therapeutic effects on metabolism related diseases such as diabetes, hyperlipidemia, or directly affect gut microbiota to regulate and ameliorate metabolic disorders (Cui et al., 2018). The gut microbiota cuts down BBR to the absorbable form of DhBBR, which converts to BBR and enters the blood after absorption in intestinal tissue, so as to improve the bioavailability of BBR (Feng et al., 2015). Studies have shown that BBR can increase its oral availability and relieve metabolic disorder by reversing the changes in the quantity, structure and composition of gut microbiota under the pathological conditions (**Table 1**). At the same time, BBR improves intestinal barrier function and reduces the inflammation of metabolism related diseases by regulating gut microbiota. In addition, BBR achieves energy balance by regulating gut microbiota dependent metabolites (such as LPS, SCFAs, BAs) and related downstream pathways. What’s more, it can improve gastrointestinal hormones and metabolic disorders by regulating bacterial-brain-gut axis (**Figure 2**).

**Table 1:** Mechanism of berberine ameliorates metabolic disorders based on gut microbiome.

Disease	Subjects	Dosage	Outcome	Changes in Gut Microbiota	Potential Mechanism	References
Hyperlipidemia	HFD-fed hamsters	100mg/kg/d for 6weeks	TC↓ TG↓ LDL ↓	*Bacteroides↑* *Escherichia-Shigella↑* *Bifidobacterium↑*	NR↑	Yan Wang et al. (2017a)
Hyperlipidemia	B6 mice	40mg/kg/d for 35 days	TC↓ TG↓ LDL ↓ TBA ↓ LPS↓ Weight↓	*A.muciniphila↑* *Sporobacter termitidis↑* *Alcaligenes faecalis↑* *Escherichia coli↓* *Desulfovibrio↓* *Parabacteroides distasonis↓*	mucus ↑ SCFAs↓	Kai He et al. (He et al., 2016a)
T2DM	db/db mice	100mg/kg/d for 55 days	FBG ↓ HbA1c ↓	*Verrucomicrobia↑* *A.muciniphila↑* *Saccharibacteria↓* *Deferribacteres↓* *Actinobacteria↓* *Firmicutes↓*	mucin-2↓	Cai Na Li et al. (Li et al., 2020)
Obesity and Insulin Resistance	HFD-fed rats	100mg/kg/d for 8 weeks	FBG↓ FINS↓ HOMA-IR↓	*Blautia ↑* *Allobaculum↑*	LBP↓	Xu Zhang el al (Zhang et al., 2012).
T2DM	KKAy Mice	100mg/kg/d for 8 weeks	HbA1c↓ HOMA-IR↓	*Vibrio desulfuricus↓* *Enterobacter↓*	LPS↓	Hui Cao et al. (Cao et al., 2020)
Insulin Resistance	HFD-fed rats	200mg/kg/d for 8 weeks	TG↓ LDL ↓ FBG↓ insulin resistance↓	*Bifidobacterium↑* *Escherichia coli↑*	LPS↓	Liu, D., et al. (Liu et al., 2018)
T2DM	db/db mice	136.5mg/kg/d for 19 weeks	food intake ↓, weight ↓, blood glucose↓ HbA1c↓	*Butyricimonas↑* *Coprococcus↑* *Ruminococcus↑*	SCFAs↓,LPS↓	Zhang, W., et al. (Zhang et al., 2019)
Atherosclerosis	High-Fat Diet-Fed ApoE−/− Mice	50mg/kg/d for 13 weeks; 100mg/kg/d for 13 weeks	atherosclerotic lesions ↓ TC↓ LDL ↓	*Roseburia↑* *Blautia↑* *Allobaculum↑* *Alistipes↑* *uricibacter↑*	SCFAs↓	Wu, M., et al. (Wu et al., 2020)
Hyperlipidemia	HFD-fed hamsters	50or200mg/kg/d for 2 weeks	TC↓ TG↓ LDL ↓	*phylum Firmicutes↑* *phylum Bacteroidetes↑*	BAs↑	Gu, S., et al. (Li et al., 2020)
hyperglycemia	db/db mice	210mg/kg for 4 weeks	Weight HbA1c↓ TG↓ LDL-c↓ FFA↓	*bacteroideae* *Clostridium*,	BAs↑	Li, M., et al. (Debose-Boyd and Ye, 2018)

This table lists the effects of BBR on laboratory indicators and gut microbiota with different doses in metabolic diseases, and the main potential mechanisms of BBR.

### 4.1 Increasing of NR-producing bacteria to promote the utilization of BBR

The oral bioavailability of Berberine is low (< 1%) as a result of poor aqueous solubility, the molecular structure of berberine prevents its rapid absorption from the intestine (Habtemariam, 2020). The extremely low plasma concentrations following oral administration of BBR in experimental or clinical settings are not sufficient to achieve the effects observed in *in vitro* experiments, which presents challenges in explaining its excellent and diverse pharmacological effects in clinical trials (Tan et al., 2013; Liu et al., 2016). Consequently, the investigators try to find out solutions in order to solve the problem of poor bioavailability (Liu et al., 2010). Previous researches have shown that gut microbiota participates in the metabolism of most oral drugs *in vivo* both directly and indirectly (Zhang et al., 2020). Some chemical components have been metabolized before the first-pass effect of liver, and the activity or toxicity of drugs can be significantly changed, affecting the exertion of drug efficacy (Sousa et al., 2008; Possemiers et al., 2011). Efficacy of intestinal microbiota in drug action remains underappreciated as before. There is increasing evidence that gut microbiota plays an important role in treatment through interaction with drugs. It contains a variety of metabolic enzymes, which can trigger a series of drug metabolic reactions and embodied the important role of gut bacteria for oral absorption of BBR (Xu et al., 2017).

Nitroreductases (NRs) are bacterial enzymes that reduce nitro-containing compounds which play key roles in BBR intestinal absorption. It has been found that various types of nitroreductase producing bacteria, such as *Enterobacter*, can produce nitroreductase to catalyze the reduction reaction of BBR to Dihydroberberine (DhBBR), which is a key bacterium to promote the intestinal absorption of BBR,NRs turns BBR into its absorbable form (DhBBR), which has highly polar and easily absorbed into the blood, and it is rapidly converted into BBR by oxidation after entering the blood. Studies have confirmed that the absorption rate of DhBBR is 5-fold correspond to BBR in animals (Direct, 2002). Some studies have also shown that after the increase of NR producing bacteria, the oral utilization and clinical efficacy of BBR can be improved. The experiment has shown that increasing fecal NR activity by approximately 10% in the gut microbiota can increase blood BBR concentrations by 65-70% in both animal experiments and human studies, suggesting that bacterial NR has a highly efficient function in converting BBR to DhBBR (Wang et al., 2017a).

Previous studies have shown that the elevation of fecal NR caused by HFD was due to an increased proportion of NR-producing bacteria in the gut or their increased activity. The bioavailability of BBR (100 mg/kg/d) in HFD-fed hamsters was higher than in normal chow-fed hamsters when administered orally. HFD caused increased mortality in the abundance of *Enterobacter spp* in mice, and BBR decreased blood lipids (such as TC, TG, LDL) in the HFD-fed hamsters, but did not play a role in those who keep with general diet (Wang et al., 2017a). In contrast, another researcher used antibiotics to reduce gut microbiota as a control group, inhibitory effect of oral treatment on gut bacteria in mice with type 2 diabetes decreased the BBR-to-DhBBR conversion and the concentration of BBR in the blood, at the same time, the lipid-lowering and hypoglycemic effects of BBR were reduced (Feng et al., 2015).Clinical study showed that NR activity in feces was higher in hyperlipidemia patients than in healthy subjects. Correlation analysis also showed that blood BBR was positively correlated with fecal NR activity. Therefore, it is reasonable to suggest that fecal NR activity is one of the effective biomarkers of BBR in the treatment of hyperlipidemia (Wang et al., 2017a).

Therefore, it is believed that HFD increases the proportion of NR producers in the gut bacterial community and/or the NR activity in bacteria. Studies showed an increase in the NR-producing bacteria in the gut microbiota community with *Bacteroides (* Schapiro et al., 2004), *Escherichia-Shigella (* Fu et al., 2007), and *Bifidobacterium (* Kinouchi et al., 1982), whose growth rate were the the highest among the top 50 bacterial strains. Moreover, Ru Feng et al. (2018) studied the pharmacokinetics of gut microbiota modulating BBR (50 mg/kg/d) and active metabolites in Beagle Dogs, as a result, an increase in the number of bacteria producing nitroreductase was observed in *Escherichia–Shigella* and *Bacteroides*.

Increasing of NR-producing bacteria was speculated to be one of the mechanisms of BBR improving metabolism. Nitroreductase activity is not only a biomarker to predict the therapeutic effect of BBR, but also an important target to improve the efficacy of BBR.

### 4.2 Increasing of mucin-degrading-producing bacteria to strengthen the intestinal barrier function

Intestinal barrier function is closely related to the occurrence and development of metabolic diseases (Scheithauer et al., 2020). T2DM is well known as a metabolic disease with low level chronic inflammation. Mucin-2 is a large glycoprotein that maintains intestinal balance by forming a physical barrier between the intestinal contents and the epithelium which is a very important glycoprotein (Paone and Cani, 2020). Thass CA (Thaiss et al., 2018) firmly believed glucose as an orchestrator of intestinal barrier function. Hyperglycemia destroys the integrity and balance of intestinal epithelial cells, interferes with the function of tight junction protein and adhesion protein between intestinal epithelial cells, leads to intestinal barrier dysfunction, increases the spread of intestinal infection, and then aggravates metabolic disorder. BBR can ameliorate endoplasmic‐reticulum stress and reduce apoptosis of goblet cells by reducing the expression of mucin‐2. Therefore, BBR has the effect of strengthening intestinal barrier.

*Akkermansia muciniphila (A.muciniphila)* has been authenticated as a mucin-degrading in the mucus layer, which has been proved to prevent the development of obesity and associated complications (Plovier et al., 2017). *A.muciniphila* treatment reversed metabolic disturbances caused by taking a high-fat diet, such as metabolic endotoxemia, adipose tissue inflammation, increased fat mass and insulin resistance in humans (Everard et al., 2013). Surprisingly, this bacterium is called a mucus degrader, and adding *A.muciniphila* increases the number of goblet cells and the production of antimicrobial peptides, which stimulated mucus production (Paone and Cani, 2020). *A.muciniphila* plays an active role in improving host metabolism and its abundance is often inversely correlated with metabolic disturbances in most preclinical and clinical studies (Zhou and Zhang, 2019), some studies made known a negative correlation among *A. muciniphila* and markers associated with metabolic disorders (Remely et al., 2015). A study has also shown that he decrease of circulating endotoxin levels mediated by *A muciniphila* can be attributed to the induction of intestinal expression of tight junction proteins (occult band protein-1 and occludin), thus reversing the increase of intestinal permeability induced by Western diet (Li et al., 2016). After BBR intervention, the abundance of *A.muciniphila* in B6 mice induced by high-fat diet was 19.1 times higher than that in the control group (He et al., 2016a). A feature of Diabetic rats fed with high fat and high-carbonhydrate diet were proinflammatory intestinal changes, altered gut-derived hormones, and what’s more, Intestinal permeability can be increased by 2.77-fold than rats on a regular diet. However, BBR treatment can significantly reverse the above changes, reduce the inflammatory changes of intestinal immune system and reduce the damage of intestinal barrier. Besides, high concentration of BBR treatment can reduce the intestinal permeability of diabetic rats by 27.5%. The researchers speculate that BBR seems to protect the integrity of intestinal barrier by regulating the expression of ZO-1 and OCLN (He et al., 2016a). Also, BBR can significantly increasing the abundance of bacteria such as *phylum Verrucomicrobia*, especially *A.muciniphila*. To decrease the FBG levels, improve the impaired oral glucose tolerance, and ameliorate the balance of α‐ and β‐cells in diabetic db/db mice, The increased abundance of *A.muciniphila* may lead to the reduction in mucin‐2 expression in ileum (Li et al., 2020). Atherosclerosis (AS) is the main co-morbidity of metabolic syndrome, research lends credence to a possible indirect role of IL-17 in determining or favoring early (Tarantino et al., 2014). This beneficial effect of BBR was associated with the regulation of gut microbiota, particularly with increased abundance of *Akkermansia (* Yang et al., 2021).

Interestingly, Gavage of BBR multiplied the abundance of *A.muciniphila* in rats. However, it did not stimulate *A.muciniphila* growth in direct incubation, which can be seen that BBR may promote *A.muciniphila* in a host-dependent way (Dong et al., 2021). How BBR stimulates *A.muciniphila* remains further research. But it can be confirmed that BBR may regulate tight junction protein and protect the integrity of intestinal barrier by increasing mucin degrading bacteria.

### 4.3 Decreasing of LPS-producing bacteria to improve metabolic endotoxemia

Studies (Cani et al., 2007a; Cani et al., 2008) proved that metabolic endotoxemia leads to inflammatory reaction which can causes weight gain and diabetes. Endotoxin plasma lipopolysaccharide (LPS) is one of the strong virulence factors of Gram-negative bacterial species, released upon lysis by LPS producing bacteria. It plays an important role in both acute infections and chronic infections (Kallio et al., 2008). It is a major active component in the generation of toxic effects from endotoxin and an initiating factor in metabolic endotoxemia. This “metabolic endotoxemia” has been widely recognized as an important reason for triggering or promoting obesity, insulin resistance, metabolic syndrome, and ultimately leading to diabetes (Cani et al., 2007b). A high-fat diet leads to an increase in the abundance of intestinal Gram-negative bacteria, such as Proteus. A large amount of LPS produced by Proteus enters the blood because of the increase of intestinal permeability. Binding of lipopolysaccharide to the complex of CD14 and toll-like receptor 4 on the surface of innate immune cells, LPS can induce systemic inflammation, which ultimately impairs insulin sensitivity and induces insulin resistance-related metabolic disorders (Pussinen et al., 2011).

*Proteobacteria* belongs to unstable potential pro-inflammatory flora. *Enterobacter*, *Vibrio desulfuricus* and *Enterobacter cloacae* increase significantly in the state of metabolic disorder, and then cause metabolic diseases (Bai et al., 2016). The abundances of *Desulfovibrio genus* were rised in obesity and T2DM (Van Hul et al., 2018). *Desulfovibrio* is supposed to an opportunistic pathogen that can produce endotoxins. The researchers found that BBR reversed these increases and reduced the level of *Vibrio desulfuricus*, in addition, they discovered that BBR treatment reduced inflammation of KKAy mice at least in part by regulating TLR4, ERK, and p38MAPK pathways (Cao et al., 2020). Some other researches showed that BBR could reduce the abundance of Vibrio desulfuricus (Zou et al., 2015) and Enterobacter (Weglarz et al., 2007) cloacae and inhibit the production of LPS. To investigate the role of BBR-mediated modulation of gut microbiota in reducing host inflammation and ameliorating insulin resistance-related metabolic abnormalities, reviewers (Zhang et al., 2012) measured the serum concentration of LBP which is a biomarker of circulating LPS. they found that HFD induced a significant increase in serum LBP level in rats, which co-administration with BBR essentially prevented, suggesting a potential role for gut microbiota antigens in this pharmacological process. Another study (Zhang et al., 2011) showed that BBR significantly reduced intestinal injury induced by LPS injury rat model and decreased serum levels of downstream inflammatory cytokines. What’s more, it is also one of the results of this study that the combined administration of BBR can significantly prevent HFD induced systemic inflammation. A Long-term HFD altered the gut microbiota composition by reducing protective bacteria like *Bifidobacterium* and increasing gram negative bacteria like *Escherichia coli*, resulting in increased release of LPS into plasma. A study showed that BBR set-back these effects and restrained LPS-induced TLR4/TNF-α activation, leading to increased insulin receptor and insulin receptor substrate-1 expression in the liver and reduce insulin resistance (Liu et al., 2018).

Gut microbial Dysbiosis affects the integrity of the intestinal epithelium and increases blood levels of LPS (Cani and Delzenne, 2009; Tagliabue and Elli, 2013). Meanwhile, HFD induces high levels of circulating LPS, which promotes metabolic inflammation and insulin resistance (Cani et al., 2007a; Cani et al., 2007b). Demonstrated that lacking the IL-17 cytokine receptor (IL-17RA^−/−^) mice in the HFD group exhibited increased intestinal permeability, with increased levels of LPS in vat of IL-17RA^−/−^ compared with C57BL/6 wild-type (WT) mice, suggesting that LPS may negatively affect insulin signaling and aggravate insulin resistance in these mice. Through a series of experimental findings, the investigators showed the importance of the IL-17/IL-17R axis in the metabolic and immunological alterations associated with the development of obesity and metabolic syndrome, by driving intestinal neutrophil migration, limiting intestinal Dysbiosis and attenuating LPS translocation to visceral adipose tissue (VAT) (Perez et al., 2019).

An up-regulation of Th17-pattern-related cytokines such as IL-6, TNFa, IL-17 and IL-22 in the intestine and increased Th17 cell. Reported data reveal that BBR can directly suppress functions and differentiation of pro-inflammatory Th1 and Th17 cells, and indirectly decrease Th cell-mediated inflammation through modulating or suppressing other cells assisting autoreactive inflammation, such as CD4+ Foxp3+ T regulatory (Tregs), dendritic cells (DCs) and macrophages (Ehteshamfar et al., 2020). Therefore, berberine may also treat metabolic syndrome by regulating intestinal permeability, alleviating inflammatory factors entering the bloodstream, and thereby attenuating insulin resistance.

In conclusion, BBR can significantly reduce the abundance of Proteobacteria, such as *Desulfovibrio*, *Enterobacter cloacae*, and inhibit LPS production, effectively prevent serum LBP elevation (Zhang et al., 2012), regulating intestinal permeability, attenuating insulin resistance and improve metabolic endotoxemia.

### 4.4 Increasing of short-chain fatty acid (SCFA)-producing bacteria to regulate inflammatory response

To a certain extent, the occurrence and development of metabolic disorders are closely related to changes in the composition of the gut microbiota and its metabolites, such as short chain fatty acids (SCFA), can significantly affect glycolipid and energy metabolism. SCFAs, mainly produced by gut bacteria ferment carbohydrates and degrade aromatic compounds, including *acetic*, *propionic*, butyric among others, have the functions of promoting regeneration of epithelial cells (Mcnabney and Henagan, 2017), strengthening intestinal barrier function, suppressing part of the inflammatory response induced by LPS, and regulating of cells in skeletal muscle, liver, and fat to Alleviating glycemic homeostasis and insulin sensitivity (Park et al., 2015). Both animal and human researches have shown that the increase of SCFAs concentration in feces is related to weight gain, fat accumulation and insulin resistance, which may be due to the increase of SCFAs production and the decrease of SCFAs absorption (Sircana et al., 2018). SCFAs work as a mediator between gut microbiota, they have the potential to improve glucose homeostasis and insulin sensitivity in patients with T2DM, and in the setting of pancreatic dysfunction, they can regulate pancreatic insulin and glucagon secretion through GLP1 augmentation in pancreatic dysfunction (Mandaliya and Seshadri, 2019). Among these, the main energy of colon cells comes from butyric acid, increasing satiety and reducing food intake (Archer et al., 2004), and is also a regulator of inflammation, modulating chronic inflammation by activating anti-inflammatory Treg cells and inhibiting pro-inflammatory cytokine and chemokine response pathways, while modulating tight junction protein expression to adjust epithelial barrier function and intestinal permeability (Wei et al., 2018).However, a high-fat diet caused dysregulation of SCFAs producing bacteria structure and decreased abundance of SCFAs producing bacteria, reducing the distribution of SCFAs in the intestine and inhibiting them from exerting normal physiological functions, result in the body occurrence a series of metabolic related diseases (Zhang et al., 2015).

Reports indicated that BBR was provided with an effect on anti-diabetic by regulating short-chain fatty acids (SCFAs). BBR intervention changed the intestinal microflora of db/db mice, increased the number of SCFA producing bacteria (such as *Butyricimonas, Coprococcus, Ruminococcus*), reduced body weight, blood sugar level and intestinal inflammation in db/db mice (Zhang et al., 2019).Regarding the way BBR works in the body, current research believes that there are two aspects: one is the direct effect of circulating BBR, and the other is the indirect effect of butyrate through the intestinal microorganism, increased abundance of *Escherichia-Shigella*, *Clostridium sensu stricto 1*, and *Bacteroides* may be one of the reasons for the increase of butyrate production (Wang et al., 2017b). The anti-atherosclerotic effect of BBR is also related to changes in composition and functions of gut microbiota. Wu, M et al. (Wu et al., 2020) studied the effects of berberine on atherosclerosis and gut microbiota regulation in ApoE (-/-) mice fed a high-fat diet, after treatment with BBR, atherosclerotic lesions was decreased and it significantly reduces total cholesterol, and very low-density lipoprotein cholesterol levels. In detail, BBR enriched the abundance o*f Turicibacter*, *Alistipes, Roseburia, Allobaculum*, and *Blautia*, and changed the abundance of *Bilophila*. These microbiota showed good anti-inflammatory effects, which are related to the production of SCFAs and significantly reduce pro-inflammatory cytokines (including TNF)- α^6^ IL-1 β And IL-6), they believe that reducing inflammation may be an important mechanism for the reduction of atherosclerosis in HFD-fed mice treated with BBR. After treated with BBR, Xu, X., et al (Xu et al., 2020) found *Clostridium XIVa, Faecalibacterium, Ruminococcus2, Coprococcus, Dorea, Butyricicoccus*, and *Roseburia* were markedly enriched in the gut microbiota. These bacteria are butyric acid producing bacteria and play a beneficial role in the host (Koh et al., 2016). In addition, increased production of fecal SCFAs was also detected in Xu’s study. *Butyricoccus, Allobaculum, Phascolarctobacterium, Blautia* and *Bacteriodes* were markedly increased by BBR in the high-fat diet-induced rats as bacteria that can produce SCFAs (Zhang et al., 2015). The efficacy was evaluated after 7 days of BBR treatment in beagle dogs, Feng, R et al. (Feng et al., 2018) found that the abundance of seven butyrate-producing genera increased than before. *Escherichia-Shigella*, *Clostridium sensu stricto 1*, *Megamonas*, *Bacteroides*, *Ruminococcus* and *Blautia* could produce butyate. That is to say BBR regulates metabolism by increasing the abundance of butyric acid producing bacteria. However, experiments in humans have also shown that BBR reduces acetate and propionate production but has no effect on butyrate levels, and that, at the same time, the use of BBR increases the abundance of *Faecalibacterium* and decreases the abundance of *Bifidobacterium*, *Streptococcus* and *Enterococcus (* Fu et al., 2022).Treatment with berberine in combination with *Bifidobacterium* could increase the abundance of beneficial bacteria in the intestine of diabetic patients and achieve better glucose lowering effect because of the inhibitory effect of berberine on *Bifidobacterium (* Ming et al., 2021).

### 4.5 Increasing of BAs-decomposing bacteria to regulate BAs-signal

Bile acids (BAs) are well known to be important metabolic and inflammatory signaling molecules that modulate lipid and energy-related nuclear hormone receptors, including farnesoid X receptor (FXR) and transmembrane G protein-coupled receptor 5 (TGR5 or GPBAR1) (Fiorucci et al., 2010). Bile acid synthesis is the main pathway of cholesterol excretion *in vivo*. Bile acids can activate TGR5 and FXR receptors, regulate blood glucose, increase glycogen synthesis, inhibit liver glycogen synthesis, protect islet cell function and maintain blood glucose homeostasis (Li and Chiang, 2014). BBR can increase the abundance of bacteria that promote the decomposition of bound bile acids and enhance the expression of bile acid receptors FXR and TGR5. BBR is considered to be an agonist of FXR and TGR5 and regulate bile acid signal and function (Han et al., 2017). In addition, bile acids have antibacterial properties, inhibit the growth of bacteria in the intestine and form a strong selective pressure on the intestinal flora. At the same time, bile acids are modified and regulated by microorganisms in the intestine (Fiorucci and Distrutti, 2015).

Research showed that BBR can significantly affect cholesterol metabolism and/or bile acid biosynthesis, suggesting that the circulating of bile acids is related to the lipid-lowering function of BBR. Previous reports have indicated that BBR treatment can increase the transforming of cholesterol to bile acid and reduce the level of cholesterol in the blood (Sun et al., 2017), and it affects the metabolism of hepatic lipids and cholesterol by increasing bile acid excretion into the large intestine (Mcdonald et al., 2012). *In vitro* studies on bile acid metabolism in the gut also showed that BBR could significantly reduce bile acid catabolism by gut microbiota in HFD hamsters. Meanwhile, Researchers predicted that BBR significantly increased CYP7A1 and CYP27A1 expression. The regulation of adipose genetic expression by insulin and fatty acids is mainly mediated by transcriptional factor, for instance SREBPs (Debose-Boyd and Ye, 2018). CYP7A1 is considered to be the classical rate-limiting enzyme for the conversion of cholesterol to bile acids. Meanwhile, this study found that BBR treatment can promote the expression of CYP7A1 in the liver of obese mice. It can be seen that the hypolipidemic effect of BBR may be related to its up-regulation of SREBP2 and CYP7A1 expression and promotion of bile acid metabolism (Feng et al., 2018).. Guo Y. et al. (Guo et al., 2016) found that BBR can significantly improve the content of BAs in serum, which is realized as an increase in primary BAs but a decrease in secondary BAs. *Bacteroides* were also observed to be enriched in the terminal ileum and large intestine of BBR-treated mice. A study showed that berberine compound increased the relative abundance of *Proteobacteria* and decreased *Firmicutes* and TM7 enrichment, which accelerate conversion of primary bile acid cholic acid (CA) into secondary bile acid deoxycholic acid (DCA) in ob/ob mice, and DCA upregulated colonic TGR5 expression and GLP secretion, thus acting as a hypoglycemic agent (Li et al., 2020). A randomized, double-blind, placebo-controlled clinical trial in 20 medical centers in China showed that BBR is mediated by the inhibition of DCA biotransformation by *Ruminococcus bromii (* Zhang et al., 2020).

The *phylum Firmicutes* have higher bile salt hydrolase (BSH) activity than the *phylum Bacteroidetes* in the intestinal microbiota, and the latter is only active against taurine-conjugated bile acids. The study distinctly shows that BBR remarkably increased the *Firmicutes/Bacteroidetes* ratio and suggests that this is one of the mechanisms of its induced serum free bile acid increase and lipid lowering effect (Gu et al., 2015).. Furthermore, there are other studies showing that bile acids induce transcriptional changes in low-abundance bile acid metabolizing bacteria, including *C. scindens (* Devendran et al., 2019), *C. hylemonae*, and *C. hiranonis* that are capable of turning taurine-conjugated bile acids into unconjugated bile acids and secondary bile acids such as ursodeoxycholic acid and lithocholic acid (Ridlon et al., 2020). BBR significantly alter gut microbial-bile acid metabolite interactions. Wolf, P.G., et al. (Wolf et al., 2021) found that sulfated bile acids were strongly associated with *C. hiranonis*, *C. hylemonae* and *Bacteroidetes spp*, which is positively correlated with the first two and negatively correlated with the last one. Also, BBR treatment increased cecal bile acid concentrations and up-regulated Na+/H+ antiporter, cell wall synthesis/repair, promote carbohydrate and amino acid metabolism. Studies have shown that the level of circulating bile acids in patients with metabolic disorders increases. BBR can increase some beneficial bacteria with benzene sulfonyl hydrazine activity, for instance *Bacteroides*, *Bifidobacterium*, *Lactobacillus* and *Clostridium*, promote the decomposition of bound bile acids and strengthen their excretion through the intestine. Lactobacillus converts primary bile acids into secondary bile acids through decarboxylation (Vincent et al., 2013). Overall, the mechanisms by which BBR alters the gut microbiome are related to its choleretic effects and are dose related.

### 4.6 Regulating microbiota-gut-brain axis to improve gastrointestinal hormone expression

The complex interaction between the brain and the gut is called the brain-gut axis, reflects the bidirectional interaction between the brain and the gut. And it is regulated by the gut microbiota, as well as mediated by brain-derived and gut derived hormones and peptides. microbiota-gut-brain axis involving many important factors such as hormones, nutrients and afferent/efferent regulatory autonomous neural pathways. A variety of physiological processes are involved at the same time, including satiety, regulation of metabolism, hormone secretion and sensitivity (especially insulin sensitivity), and bone metabolism (Romijn et al., 2008).. Recently, researchers have found that the disturbance of the gut-brain axis is closely related to the occurrence and development of metabolic diseases and exerts its effects through the hormone signaling pathway (Burokas et al., 2015). Studies have demonstrated that gastrointestinal hormones, such as ghrelin, orexin, glucagon-like peptide-1 (GLP-1), and leptin, can regulate feeding behavior, energy homeostasis, etc. (Cameron and Doucet, 2007; De Silva and Bloom, 2012).Numerous studies have shown that GLP-1 receptor agonists have good clinical efficacy in the treatment of diabetes and obesity (Han et al., 2017). Studies have shown that GLP-1 promotes beta-cell neogenesis and satiety, reduces glucagon secretion, delays gastric emptying, and increases peripheral glucose disposal (Donnelly, 2012). Drugs activating the GLP-1 receptor are also beneficial in the management of another current epidemic, namely nonalcoholic fatty liver disease (NAFLD) (Paternoster et al., 2019). Changes in gut hormones, containing increases in GLP-1 might have a role in induction and long-term maintenance of weight loss (Finelli et al., 2014). Consequently, the microbiota-gut-brain axis is a potential target for metabolic disease treatment and one of the hotspots for future research.

Studies have shown that BBR decreases the variousness of intestinal flora, increases the proportion of *Bacteroidetes* and *Firmicutes*, further increases the expression of serum GLP-1, GLP-2 (Wang et al., 2021), PYY, GIP and ghrelin (Sun et al., 2016), provides evidence that BBR treatment can inhibit microbiota diversity, elevate plasma GLP-1 and orexin-a, and upregulate hypothalamic GLP-1 receptor expression, which has beneficial effects on various metabolic disorders such as insulin resistance, obesity and obesity, thereby Induces regulation of the gut-brain axis of the microbiota (Sun et al., 2016). Previous studies (Lu et al., 2009; Yu et al., 2010) showed that that BBR increased the number of L-cells and the mRNA expression levels of proglucagon in the ileum, while promoting GLP-1 secretion in normal and diabetic rats. Other studies have also shown (Xu et al., 2017) BBR significantly increased the levels of plasma GLP-1 and GLP-2 in portal vein, as well as the number of L-cells in proximal colon and the mRNA expression level of preluganin. Some studies have linked the intestinal microbiota to the intestinal endocrine system (Cani et al., 2013). Butyrate may improve metabolism *via* gut-brain axis signaling (De Vadder et al., 2014). The results showed that after BBR intervention, the number of L-cells was positively correlated with the abundance of *acteria, anaerobes, cholephila and oscillibacter*. Studies indicated that *Akkermansia muciniphila* could significantly increase GLP-1 release from colonic L cells (Everard et al., 2011; Hansen et al., 2011), this study confirmed that BBR increased the abundance of akman bacteria and decreased the abundance of Lactobacillus, which seems to be related to the increase of the number of L cells and the intestinal endocrine peptides secreted by L cells. In summary, regulation of glucose and gut hormone levels by BBR has a lot to do with modulating the composition of the gut microbiome. There are few studies on BBR in the treatment of metabolic diseases by regulating microbiota-gut-brain axis, for its potential effectiveness, this mechanism is worthy of further study.

## 5. Discussion

Previous studies have shown that metabolic disorders significantly affect the structure and function of gut microbiome. In addition, gut microbiome can affect body metabolism by producing metabolites such as short chain fatty acids and succinic acid, which increases the abundance of beneficial bacteria and decreases the abundance of pathogenic bacteria. Therefore, this study focused on the abundance of beneficial and pathogenic bacteria after BBR intervention, and summarized the mechanism of BBR on metabolic diseases. In recent years, with the continuous unraveling of the interactions between gut microbiome and organismal metabolism, relevant intervention mechanisms including the regulation of short chain fatty acids, bile acid metabolic pathways, LPS/TLR4 signaling have been gradually studied (Harsch and Konturek, 2018). However, based on fecal microbiota transplant (FMT), metagene Association, and other techniques, it remains to be explored from dissecting the overall structural changes in gut microbiome to the specific effects of a single genus on organism metabolism. In addition, because the complexity of the interaction mechanism between gut microbiome and body metabolism leads to a causality still not fully clear and needs to be elucidated in more rational experimental designs and research approaches (Fischbach, 2018), it is hoped that gut microbiome will become more widely used in clinical practice as a therapeutic target for metabolic diseases.

Numerous studies have shown that BBR modulates the gut microbiome, and its effects on modulating metabolic function are influenced by the gut microbiota. For example, BBR improves lipid levels in HFD-fed rats, whereas it does not exert a fat regulating effect in normal rats. Further study revealed that the abundance of Nitroreductases producing bacteria increased under the metabolic disorder state, which can produce more Nitroreductases, catalyze the conversion of BBR to Dihydroberberine and promote the intestinal absorption of BBR. So that the metabolic effect of BBR improvement is dependent on the structure and function of gut microbiota. This also reveals, to some extent, the biological basis for the interindividual differences in the efficacy of BBR in regulating body metabolism in the clinic (Wang et al., 2017a).

After extensive literature reading and summarizing, we believe that in the exploration of BBR based on the mechanism related to gut microbiome regulating organism metabolism, it still needs to be combined with individual differences in gut microbiome and well-established experimental protocols to seek the best intervention mode and intervention dosage for clinical application. Then, different conclusions remain to be reported about the structural changes of intestinal flora caused by Berberine: Some believe that the abundance of *Akkermansia* increased after BBR intervention, But while others believe that BBR could not increase the abundance of *Akkermansia* alone. In addition, some of the mechanisms by which berberine regulates the structural changes of the flora remain to be explored, for instance, how BBR stimulates *A.muciniphila* remains further research. Therefore, not only the results of the pre-existing studies need to be validated, but the reasons leading to the discrepancy of the results should be further analyzed.

This review summarizes the mechanism of berberine improving metabolic disorder based on gut microbiome through a combing of literatures. It was found that BBR had a clear effect on altering the abundance of specific bacteria in the gut and modulating the structural function of the gut microbiome as a whole. Through strengthening intestinal barrier function, attenuating metabolic endotoxemia, modulating systemic inflammatory responses, bile acid signaling, and the Microbiota-Gut-Brain axis, among other pathways, in order to exert its effect of improving metabolic disorders. As more studies clarified the mechanisms and characteristics of BBR in ameliorating metabolic disorders by regulating gut microbiome, it provided a new target and research direction for the diagnosis and treatment practice of metabolic diseases in the future clinic. It is worth mentioning that BBR, as a widely used compound in clinic, both in animal experiments and clinical studies, more and more studies have focused on the effects of different doses of BBR on the gut microbiome, and on the basis of confirming its efficacy, dose studies will provide guidance for further exploring the mechanism of action of BBR.

## Author contributions

HW and QZ designed and drafted the manuscript. HZ and ZG conceived the manuscript. CG edited and added valuable insights into the manuscript. All authors approved the final manuscript and agreed to be accountable for all aspects of the work.

## Funding

This work was supported by the National Natural Science Foundation of China (Grant Nos. 81973837, 81704067 and 81430097), China. And the Strategic Priority Research Program of Chinese Academy of Sciences (Grant No. XDB29020000), China.

## Conflict of interest

The authors declare that the research was conducted in the absence of any commercial or financial relationships that could be construed as a potential conflict of interest.

## Publisher’s note

All claims expressed in this article are solely those of the authors and do not necessarily represent those of their affiliated organizations, or those of the publisher, the editors and the reviewers. Any product that may be evaluated in this article, or claim that may be made by its manufacturer, is not guaranteed or endorsed by the publisher.
